# Global transcriptome analysis of the aphelid *Paraphelidium tribonemae* supports the phagotrophic origin of fungi

**DOI:** 10.1038/s42003-018-0235-z

**Published:** 2018-12-19

**Authors:** Guifré Torruella, Xavier Grau-Bové, David Moreira, Sergey A. Karpov, John A. Burns, Arnau Sebé-Pedrós, Eckhard Völcker, Purificación López-García

**Affiliations:** 1 Unité d’Ecologie, Systématique et Evolution, CNRS, Université Paris-Sud, Université Paris-Saclay, AgroParisTech, 91400 Orsay, France; 2 Institut de Biologia Evolutiva, Universitat Pompeu Fabra-CSIC, 08003 Barcelona, Catalonia Spain; 3 Zoological Institute, Russian Academy of Sciences and St. Petersburg State University, St. Petersburg, Russian Federation 199134; 4 Sackler Institute for Comparative Genomics and Division of Invertebrate Zoology, American Museum of Natural History, New York, 10024-5192 NY USA; 5 Weizmann Institute of Science, Rehovot, 7610001 Israel; 6 Penard Laboratory, Cape Town, 7708 South Africa

**Keywords:** Evolution, Phylogenetics

## Abstract

Aphelids are little-known phagotrophic parasites of algae whose life cycle and morphology resemble those of the parasitic rozellids (Cryptomycota, Rozellomycota). In previous phylogenetic analyses of RNA polymerase and rRNA genes, aphelids, rozellids and Microsporidia (parasites of animals) formed a clade, named Opisthosporidia, which appeared as the sister group to Fungi. However, the statistical support for the Opisthosporidia was always moderate. Here, we generated full life-cycle transcriptome data for the aphelid species *Paraphelidium tribonemae*. In-depth multi-gene phylogenomic analyses using several protein datasets place this aphelid as the closest relative of fungi to the exclusion of rozellids and Microsporidia. In contrast with the comparatively reduced *Rozella allomycis* genome, we infer a rich, free-living-like aphelid proteome, with a metabolism similar to fungi, including cellulases likely involved in algal cell-wall penetration and enzymes involved in chitin biosynthesis. Our results suggest that fungi evolved from complex aphelid-like ancestors that lost phagotrophy and became osmotrophic.

## Abstract (web summary)

Guifré Torruella et al. present the first transcriptome data of an aphelid species, which multi-gene phylogenomic analyses place as the closest relative of fungi. They are able to infer a free-living-like aphelid proteome and suggest that fungi evolved through the loss of phagotrophy from aphelid-like ancestors.

## Introduction

Fungi are an extremely ecologically successful group of organisms involved in the saprophytic degradation of organics, with some parasitic species. They feed by osmotrophy or direct uptake of dissolved organics through their chitin cell-wall^1^. Traditionally, fungi encompassed several lineages of filamentous species, most of which belong to the Dikarya (Ascomycota and Basidiomycota), and a group of flagellated species known as chytrids (Chytridiomycota)^2^. The first molecular phylogenetic analyses based on one or a few genes confirmed the monophyly of Dikarya but also revealed that some deep-branching fungal lineages were not monophyletic, notably the chytrids and the zygomycetes^2,3^. In parallel, environmental studies have uncovered a wide diversity of sequences belonging to new lineages of unicellular eukaryotes branching at the base of the classical fungi. One of those lineages (known by several names: Rozellida, Cryptomycota, Rozellomycota) includes the flagellated phagotrophic unicellular parasite *Rozella allomycis*, which branches as a sister lineage to classical fungi in phylogenetic trees^4,5^. Another group corresponds to the Microsporidia, well-known fast-evolving parasites, whose deep phylogenetic branches are progressively being populated by new, slowly-evolving species^6^. The third clade of organisms that appear to relate to these lineages is the aphelids (Aphelida)^7^. While some authors include all of these protist lineages as fungi (fungi *sensu lato*)^8,9^, others do not^7^. Delimiting what defines a fungus is not easy, especially when the phylogenetic relationships among fungi-related protists remain undetermined^1^. Here, we refer to fungi as encompassing the classical core fungi (filamentous and chytrid fungi), which feed by osmotrophy through chitinous cell walls.

Aphelids constitute a group of diverse, yet poorly known, parasites of algae^7,10,11^. Their life cycle and morphology resemble those of zoosporic fungi (chytrids) and rozellids (Cryptomycota/Rozellosporidia), another species-rich group of parasites of fungi and oomycetes^4,5^. Unlike fungi, which are osmotrophs, aphelids and rozellids are phagotrophs, feeding on the host’s cytoplasm. Combined RNA polymerase and rRNA gene trees^12^ suggested that aphelids and rozellids relate to Microsporidia, extremely reduced parasites with remnant mitochondria unable to perform phagotrophy^13^. Accordingly, aphelids, rozellids and Microsporidia were proposed to be monophyletic and form a sister group to fungi called Opisthosporidia^7^. However, the limited phylogenetic signal of those genes combined with fast-evolving sequences from microsporidians have resulted in incongruent tree topologies, showing either rozellids^12,14^ or aphelids^15^ as the earliest-branching lineages of Opisthosporidia. Furthermore, the support for the monophyly of Opisthosporidia was always moderate, leaving the relative order of emergence of the highly diverse fungal relatives unresolved. To improve the phylogenetic signal for an accurate placement of aphelids in the opisthokont branch containing fungi, nucleariids, rozellids, and Microsporidia (usually referred to as Holomycota), we have generated the first full life-cycle transcriptome data for one aphelid species, *Paraphelidium tribonemae*, to our knowledge^11^. Phylogenomic analyses of this almost-complete transcriptome including, in particular, data from the *Rozella allomycis* genome^16^, show that Opisthosporidia are paraphyletic and that *P. tribonemae* branches as the sister lineage to Fungi. Comparison of gene sets involved in metabolism and major cellular functions strongly suggests that fungi evolved from complex aphelid-like ancestors that were phagotrophic.

## Results

### Aphelids occupy a deep pivotal position and are the sister group to fungi

**Fig. 1:**
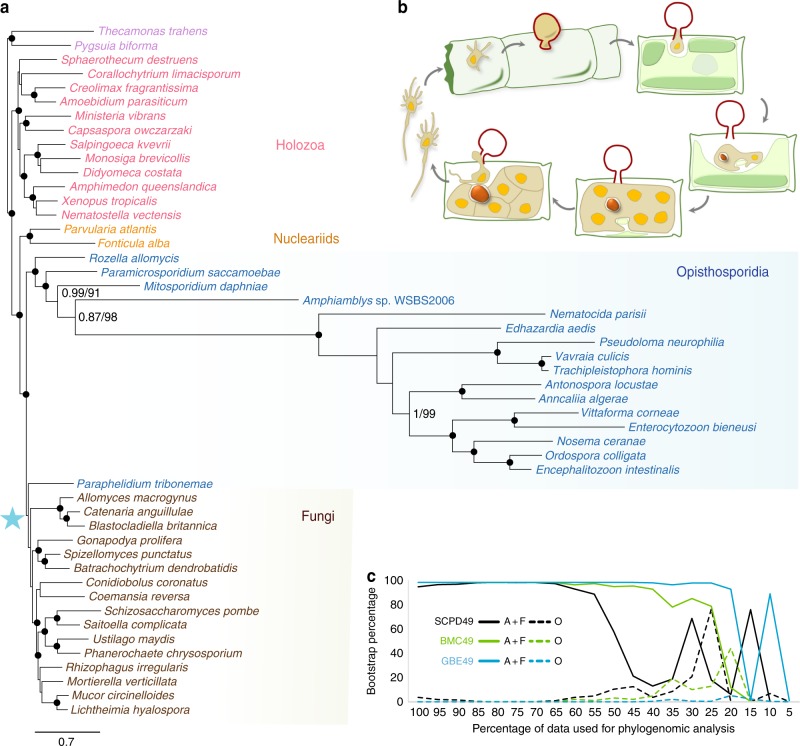
Phylogenomic analyses and cell cycle of the aphelid *Paraphelidium tribonemae*. **a** Bayesian phylogenetic tree based on single-copy protein domains for 49 species (SCPD49) inferred using a CAT-Poisson model. Statistical supports indicated at some crucial nodes correspond, from left to right, to PhyloBayes Bayesian posterior probabilities and IQ-TREE ML ultrafast-bootstrap support using the C60 model. Branches with maximum support values (pp = 1 and ubs = 100%) are indicated by black circles. The support for the monophyly of *Paraphelidium* and fungi (blue star) using the three datasets (SCPD, BMC and GBE) with the 49 species and after removing the fastest-evolving microsporidian sequences (36 species) is shown in Table 1. **b** Schematic cell cycle of *P. tribonemae*. Briefly, infecting cysts (red wall), deliver an ameboid trophont to an algal filament cell via an infection tube; the trophont engulfs the algal cytoplasm by phagocytosis, leaving a growing residual body (dark red particle); after nuclear and cell division, a mature sporangium releases amoeboflagellated zoospores (occasionally ameboid only) that get out the algal cell wall and close the life cycle^11^. **c** Evolution of IQ-TREE ML ubs support for the monophyly of aphelids and fungi (A + F) and the monophyly of Opisthosporidia (O) as a function of the proportion of fast-evolving sites removed from the dataset. All the phylogenomic trees can be seen in Supplementary Fig. 1

To generate the aphelid transcriptome and because *P. tribonemae* has a complex life cycle (Fig. 1), we maximized transcript recovery by constructing two cDNA libraries corresponding to young and old enrichment cultures of its host, the yellow-green alga *Tribonema gayanum*, infected with the aphelid. Accordingly, transcripts corresponding to zoospores, infective cysts, trophonts, plasmodia, sporangia and sporocysts were represented^11^. After paired-end Illumina HiSeq2500 sequencing, we assembled a metatranscriptome of 68,130 contigs corresponding to the aphelid, its host and bacterial contaminants. After a sequential process of supervised cleaning, including comparison with a newly generated transcriptome of the algal host, we obtained a final dataset of 10,439 protein sequences that were considered free of contaminants. The final predicted proteome (*Paraphelidium tribonemae* version 1.5; Supplementary Data 1) was 91.4% complete according to BUSCO^17^. We found no stop codons interrupting coding sequences. Therefore, in contrast to *Amoeboaphelidium protococcarum*, for which the TAG and TAA stop codons appear to encode glutamine^12^, *P. tribonemae* does not possess these modifications from the canonical genetic code.

**Table 1:** Statistical support for the monophyly of *Paraphelidium* and fungi (blue star in Fig. 1) in multi-gene phylogenetic trees using three datasets (SCPD, BMC, and GBE) and two sets of species including (49 species) or excluding (36 species) the fastest-evolving microsporidian sequences

Dataset	49 Species	36 Species
SCPD	0.66/96	0.88/95
BMC	1/100	1/100
GBE	0.5/100	1/100

Because resolving the phylogenetic relationships in this part of the eukaryotic tree is challenging due to the occurrence of lineages with very different evolutionary rates, notably the fast-evolving Microsporidia, we used three different datasets and carried out a battery of phylogenomic analyses including and excluding the fastest-evolving species and amino acid positions from our datasets. The three datasets have been previously used to study the phylogeny of animals and related protists (Holozoa, the sister group to Holomycota) within Opisthokonts (SCPD)^18^, Microsporidia (including highly conserved genes for this fast-evolving group, BMC)^19^, and Microsporidia and related deeper-branching lineages (maximizing the putative orthologs in the whole clade, GBE)^15^. First, we incorporated *P. tribonemae* orthologs to a dataset of 93 single-copy protein domains (SCPD dataset) previously used for phylogenomic analyses of basal-branching opisthokonts^18^, updating it with several microsporidian species including the early-branching *Mitosporidium daphniae*^20^, the metchnikovellid *Amphiamblys* sp.^15^, and the recently released genome of *Paramicrosporidium saccamoebae*^21^. We then generated Bayesian inference trees reconstructed under the CAT-Poisson mixture model^22^ and maximum likelihood (ML) trees reconstructed under C60, the closest mixture model to CAT^23^, for a taxon sampling including 49 species (22,976 positions). The two phylogenomic analyses yielded the same topology. We recovered the monophyly of all opisthokont lineages previously reported^18,24^. In contrast with previous analyses based on 18S rRNA and RNA polymerase genes^12,14^, Opisthosporidia appeared as paraphyletic and the aphelid was placed as the sister lineage to fungi (Fig. 1). However, the ML bootstrap support was moderate (ultrafast bootstrapping; 96% ubs) and the Bayesian probability was low (0.66 pp; even if the two Bayesian chains converged in terms of likelihood, the consensus topology was not distinctively supported by the other chain). To limit potential long-branch attraction artefacts derived from the inclusion of fast-evolving Microsporidia, we built a reduced dataset without the thirteen fastest-evolving microsporidian species (SCPD36, 36 species; 24,413 positions). Bayesian and ML trees again reproduced the same topology (Supplementary Fig. 1) with higher pp (0.88) and ubs (95%) values, but still not fully supporting the consensus topology (Fig. 1; Table 1).

To resolve the phylogenetic position of the aphelid with higher support, we analyzed two additional datasets previously used for phylogenomic analyses of Microsporidia with the same two sets of 49 and 36 opisthokont species. The resulting datasets were called BMC49 and BMC36 for a set of 53 protein markers^19^ which, after concatenation, yielded 15,744 and 16,840 amino acid positions, respectively; and GBE49 and GBE36 for datasets with 259 protein markers^15^, which resulted in 84,810 and 96,640 amino acids, respectively. For all datasets, Bayesian and ML trees recovered the monophyly of *Paraphelidium* and fungi (Supplementary Fig. 1) and, for the two datasets without fast-evolving Microsporidia, posterior probabilities and ML bootstrap support were maximal (Fig. 1, Table 1, Supplementary Data 2). In addition, we carried out alternative topology tests for the three datasets containing 49 species. All of the alternative topology tests supported *P. tribonemae* as the sister group to fungi (AU test *p*-value significantly excluded if <0.1), and both BMC49 and GBE49 significantly excluded the Opisthosporidia monophyly (AU *p*-value 0.0039 and 0.0000, respectively; Supplementary Data 2). Finally, to minimize systematic error in ML analyses due to the inclusion of fast-evolving sites in our protein datasets, we progressively removed the 5% fastest-evolving sites and reconstructed the phylogenomic trees. This resulted in the increase of support for the monophyly of fungi and the aphelid, which became very high (>98% ubs; see Supplementary Data 2) until 50% (SCPD dataset) or up to 80% (GBE dataset) of sites were removed, when phylogenetic signal was expected to be insufficient to resolve such deep phylogeny. Remarkably, the Opisthosporidia monophyly was never supported (Fig. 1c).

Collectively, our phylogenomic analyses support the monophyly of aphelids and fungi, although the branch joining the two groups is very short. Opisthosporidia, united by their ancestral phagotrophy, appear as paraphyletic.

### Enzymes involved in cell-wall synthesis and degradation

**Fig. 2:**
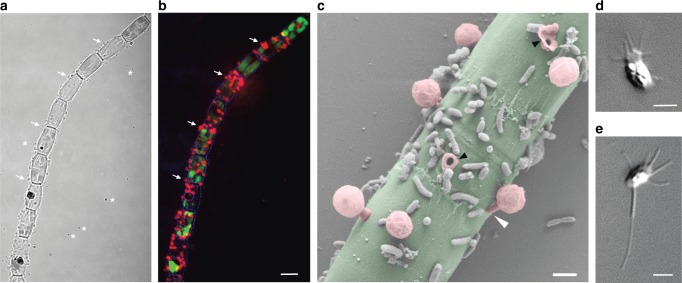
Zoospores and chitin-bearing infective cysts of *Paraphelidium tribonemae*. **a** Filament of *Tribonema gayanum* infected by *P. tribonemae* under optical microscopy. Many cysts can be seen attached to the filament surface, many (small) zoospores can be seen surrounding the filament before encystment; trophonts are present within *Tribonema* cell-wall delimited compartments. **b** The same filament as in **a** stained with fluorescent wheat germ agglutinin (WGA, red color) showing the presence of chitin in infective cysts (arrows show some examples) but not the zoospores (asterisks show some examples) under epifluorescence microscopy. **c** False-colored scanning-electron microscopy image of a filament infected by several *P. tribonemae* cysts (pedunculated rounded structures). The algal host filament is colored in green and parasite cysts in pink. Note that one cyst germ tube is penetrating the host cell by the space between the two algal cell-wall halves (white arrowhead) and two cysts are broken (black arrowheads), showing the penetration channel. **d** Ameboid zoospore (infrequent). **e** Amoeboflagellated zoospore. Scale bar: *A* = 5 µm, B–F = 1 µm. Phylogenetic trees related to chitin and other cell-wall synthesis and degradation-related enzymes are shown in Supplementary Fig. 2. Zoospore motility can be seen in Supplementary Video 1. For original fluorescent microscopy images, see Supplementary Data 7

Despite secondary losses in some fungi, the presence of chitin in cell walls was long considered a typical fungal trait^1^. However, chitin is also present in the cell wall of many other protists across the eukaryotic tree, implying that the machinery for chitin synthesis/remodeling originated prior to the radiation of fungi and other eukaryotic lineages^1^. Microsporidia and *Rozella* are also able to synthesize chitin^16,25^ but, unlike fungi, which possess chitin cell walls during the vegetative stage, they produce chitin only in cysts or resting spores^26^. Staining with fluorescently-labeled Wheat Germ Agglutinin (WGA) showed that *Paraphelidium* also possesses chitin in the wall of infecting cysts, but not in zoospores or, unlike fungi, in the vegetative stage (i.e., the trophonts) (Fig. 2a, b). In agreement with this observation, we identified homologs of chitin synthases, chitin deacetylases, chitinases and 1,3-beta-glucan synthases in the *Paraphelidum* transcriptome (Supplementary Fig. 2a–d). Specifically, we detected seven homologous sequences (including all alternative transcripts such alleles or splice variants) of division II chitin synthases^18,25,27,28^ in *Paraphelidium* corresponding to at least six distinct peptides (*Rozella* contains only four^16^). Three of them clustered with class IV chitin synthases, including Microsporidia and *Rozella* homologs (Supplementary Fig. 2a). The remaining four sequences branched within class V/VII enzymes^28^, two of them (probably corresponding to a single polypeptide) forming a deep-branching group with fungal, mostly chytrid, sequences (Supplementary Fig. 2a). Class V enzymes include a myosin motor thought to intervene in polarized fungal hyphal growth that has been hypothesized to take part in the formation of the germ tube in aphelids and rozellids^20^. Class V chitin synthases were lost in Microsporidia (with the exception of *Mitosporidium*, still retaining, like *Rozella*, one homolog), endowed instead with highly specialized polar tube extrusion mechanisms^20^. Neither spore wall nor polar tube proteins specific to Microsporidia^29^ occurred in the *Paraphelidium* transcriptome. Therefore, our data (Supplementary Fig. 2a; Supplementary Data 1 and 5) lend credit to the hypothesis that class V chitin synthases are involved in germ tube polar growth.

Among the rest of chitin-related enzymes, we identified twelve sequences (at least five different homologs) of chitin deacetylase^30,31^ (Supplementary Fig. 2b). We detected at least three class II chitinase homologs (eight total sequences), which are ancestral in opisthokonts, containing the Glyco_hydro_18 (PF00704) domain, and a class I chitinase (CTSI) with a Glyco_hydro_19 (PF00182) domain^32^. The latter included the catalytic site, an N-terminal predicted signal peptide and a transmembrane region, suggesting an extracellular chitinase activity. CTSI has a peculiar phylogenetic distribution in eukaryotes, occurring only in Viridiplantae, Fungi, Opisthosporidia and Ecdysozoa (Supplementary Fig. 2c). *Rozella* contains two homologs and Microsporidia at least one; they have N-terminal signal peptides and are predicted to localize extracellularly but lack transmembrane domains. In our phylogenetic tree, opisthosporidian sequences appeared scattered within metazoan and fungal sequences. This might be the result of hidden paralogy and/or horizontal gene transfer (HGT) (Supplementary Fig. 2c). Regardless of its evolutionary origin, aphelid CTSI might be involved in the self-degradation of resting spore and cyst wall chitin. This might happen both, during their release from chitin-containing resting spores or at the tip of the germ tube during infection, as previously suggested for *Rozella*^16^ (Fig. 1).

Although not found in chytrids, 1,3-beta-glucan is also considered an idiosyncratic fungal cell-wall component^1^. Surprisingly, we identified two 1,3-beta-glucan synthase (FKS1) homologs (four sequences), with split Glucan_synthase (PF02364) and FKS1_dom1 (PF14288) domains (fused for the phylogenetic analysis) (Supplementary Fig. 2d). The presence of FKS1, absent in *Rozella* and Microsporidia, in aphelids traces its origin back to the ancestor of fungi and aphelids.

To feed on the algal cytoplasm, aphelids need to traverse the algal cell wall but, so far, the specific penetration mechanism, whether mechanical (germ-tube penetration through the gap between the two algal cell-wall halves) or enzymatic (digestion of algal cell-wall components) was uncertain^7,11^. Scanning electron microscopy (SEM) observations showed both, clear cases of mechanical penetration between the two algal cell-wall halves, and also infecting cysts scattered on the algal cell-wall surface (Fig. 2c). SEM images additionally confirmed WGA-epifluorescence observations of multiple parasitoid cysts co-infecting the same host cell (Fig. 2a–c). Multiple infections, implying the coexistence of distinct trophonts feeding within the same algal cell-wall delimited compartment, open the intriguing possibility for aphelids to engage in sexual reproduction within the algal host. Our *Paraphelidium* transcriptome has some genes involved in meiosis  (e.g., MutS protein homolog 4), but not all (Supplementary Data 1). However, the transcriptome is not complete and, at the same time, many of the detected genes are also involved in more general recombination (e.g., meiotic recombination protein DMC1) and DNA repair (e.g., multiple helicases homologous to HFM1) processes. Therefore, whether the aphelid has the potential to carry out meiosis and whether it might indeed occur at the trophont stage will require future study. *Tribonema* cell walls contain cellulose II based on 1,6-linked glucan (alkali soluble cellulose), 1,3 and 1,4-linked xylose, 1,3-linked rhamnose and, mostly, 1,3; 1,4; and 1,6-linked glucose^33^. We performed sequence similarity searches of known fungal cellulases^34^ using the database mycoCLAP^35^, which contains functionally characterized proteins, to search for these enzymes in aphelids, followed by phylogenetic analyses. In support of an enzymatic algal cell-wall penetration, we identified various cellulases in the *Paraphelidium* transcriptome belonging to glycoside-hydrolase families GH3 and GH5. We detected three homologs of the GH3 cellulase beta-glucosidase/xylosidase^36^, which is not found in other opisthosporidians but is present in fungi, amoebozoans, several opisthokonts and other protists, as well as bacteria. Our phylogenetic analysis shows that the three aphelid sequences are most closely related to deep-branching opisthokont protists (respectively, *Capsaspora*, choanoflagellates, nucleariids) (Supplementary Fig. 2e). Additionally, we identified at least three GH5 cellulase^37^ homologs (seven sequences in total) in *P. tribonemae*, which were related to GH5 widespread in fungi (GH5_11, GH5_12 and GH5_24) (Supplementary Fig. 2f). Collectively, these observations strongly suggest that these cellulases are involved in the alga cell-wall penetration, but direct proof will only be obtained by purifying or heterologously expressing those cellulases and testing their activity in vitro.

### Primary metabolism reminiscent of free-living lifestyles

**Fig. 3:**
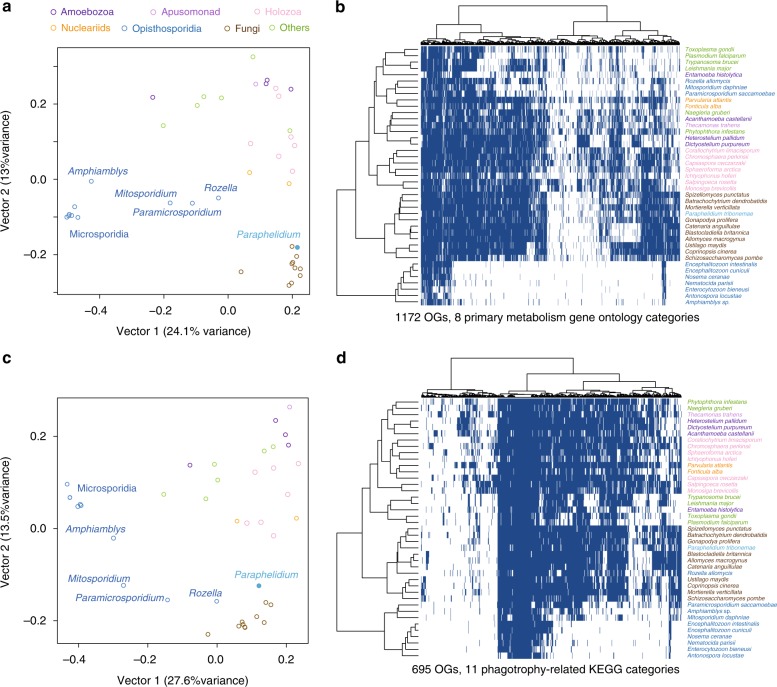
Complexity of *Paraphelidum tribonemae* metabolism and cytoskeleton-trafficking-phagotrophy-related proteome. **a** Principal coordinate analysis (PCoA) and **b** binary heat-map and species clustering based on the presence/absence of 1172 orthologous genes belonging to 8 primary metabolism Gene Onthology categories across 41 eukaryotic genomes/transcriptomes. **c** PCoA and **d** binary heat-map and species clustering based on the presence/absence of 695 KEGG orthologs related to cytoskeleton, membrane-trafficking and phagotrophy, which were selected from 11 KEGG categories. Species are color-coded according to their taxonomic assignment

Analysis of the *Rozella allomycis* genome showed that, like microsporidian parasites, it has considerably reduced metabolic capabilities^16^. To comparatively assess the metabolic potential of aphelids, we investigated the presence of orthologous groups related to eight primary metabolism categories (Gene Ontology) in the transcriptome of *Paraphelidium*, using eggNOG annotation^38^. We thus identified 1172 orthologous groups in *Paraphelidium* and a set of 41 eukaryotic species including representatives of major fungal lineages, opisthokont protists and other eukaryotic parasites (Supplementary Data 3). Based on their orthologous group distribution, we built a dissimilarity matrix that was analyzed by Principal Coordinate Analysis. The first axis clearly separated *Paraphelidium* from Microsporidia, *Mitosporidium*, *Paramicrosporidium* and *Rozella*, the latter two positioned near one another and having an intermediate position similar to other protist parasites (e.g., *Trypanosoma, Leishmania, Toxoplasma*) (Fig. 3a and Supplementary Fig. 3a). *Paraphelidium* was positioned at the same level as fungi, *Capsaspora, Corallochytrium* and *Parvularia*, along axis 1. However, axis 2 separated *Paraphelidium* and fungi from the rest of eukaryotes. These relationships were further visualized in a cluster analysis of pairwise species comparisons (Supplementary Fig. 3b). The PCoA suggested that *Paraphelidium* has a rich metabolic gene complement, which was made evident by the orthologous group presence/absence heatmap showing that aphelids have a metabolic potential fully comparable to that of (especially chytrid) fungi (Fig. 3b).

The most distinctive metabolic categories when comparing *Paraphelidium* and other Opisthosporidia were energy production and conversion, followed by amino acid, nucleotide and lipid transport and metabolism. In all metabolic categories, the aphelid clustered with fungi, and more specifically with chytrids, and sometimes with other free-living opisthokonts (e.g., nucleariids, *Capsaspora*). By contrast, *Rozella* always clustered with *Mitosporidium* and *Paramicrosporidium* either together with other Microsporidia or with other parasitic protists (Supplementary Fig. 3c–f). The only exception corresponded to *Paramicrosporidium* which, for energy production and conversion, clustered with nucleariids (Supplementary Fig. 3c), in agreement with their rich energy-related gene set^21^.

To check whether these differences between *Paraphelidium* and *Rozella* affected particular metabolic pathways, we compared the annotated proteins in the two organisms based on KEGG annotation^39^. A comparison of the two species in the KEGG general metabolic pathway map showed that, even accounting for the possibility that we missed some genes in the *Paraphelidium*’s transcriptome (e.g., mitochondrial-encoded proteins), the aphelid map contained 200 orthologous groups more than *Rozella* (548 vs. 348 orthologous groups) (Supplementary Fig. 3g). In agreement with previous observations, major differences were observed in energy production and conversion, and amino acid, nucleotide and lipid transport and metabolism. In particular, contrary to *Rozella*, which lacks most subunits of the mitochondrial electron transport chain complex I (NADH dehydrogenase; ETC-I)^16^, *Paraphelidium* possesses a practically complete ETC-I as inferred from the nuclear encoded transcripts (the *P. tribonemae* transcriptome is biased against mitochondrial transcripts, which lack polyA) (Supplementary Fig. 3h). *Paraphelidium* also possesses wide capabilities related to nucleotide (e.g., purine, uridine, and inosine biosynthesis) and amino acid (serine, threonine, methionine, lysine, ornithine, histidine, shikimate pathway) metabolism, which *Rozella* has lost (Supplementary Fig. 3i). Likewise, the aphelid has also retained pathways for phosphatidylcholine, cholesterol and fatty acid biosynthesis that were subsequently lost in *Rozella*^16^. Most carbohydrate metabolic pathways were conserved in the two species, except for the galactose synthesis and degradation that was lost in *Rozella* (Supplementary Data 3).

By contrast, compared to *Rozella*, and under the assumption that our transcriptome is rather complete, the aphelid seems to lack several enzymes involved in catecholamine biosynthesis (Supplementary Data 3). However, some of these are also absent from *Capsaspora, Monosiga, Salpingoeca*, or *Spizellomyces*. These compounds are likely involved in cell–cell communication in microbes^40^, e.g., parasite-host signaling, suggesting that they might have a role in rozellid parasitism. The aphelid seems to lack other parasite-specific proteins, such as crinkler, nucleoside H^+^-symporters or ATP/ADP-antiporters, which occur in *Rozella* and/or Microsporidia^16^.

These observations suggest that *Paraphelidium* has a complex metabolic profile, being functionally closer to free-living protists than to parasites and having affinities with fungi and, to a lesser extent, nucleariids and holozoan protists.

### Distinct and ancestral-like phagotrophy-related machinery

Like rozellids, aphelids are phagotrophs, but their global metabolism resembles that of osmotrophic fungi. What does their phagocytosis-related proteome look like? The core phagocytic molecular machinery, already present in the last eukaryotic common ancestor^41^, is involved in multiple dynamic cell processes (endocytosis, exocytosis, autophagy, protein recycling, etc.). Structurally, the phagocytic machinery encompasses various endomembrane organelles (phagosomes, lysosomes, peroxisomes, endoplasmic reticulum, Golgi apparatus), and multiple membrane-trafficking components (signaling pathways, transporters, specific enzymes, cytoskeleton elements, motors, etc.)^42^. To examine phagotrophy-related genes in *Paraphelidium* and the 41 additional eukaryotes used for comparison, we built a presence/absence matrix of 695 KEGG orthologs from 11 functional categories (5 KEGG maps and 6 BRITE categories) that aimed at including all necessary proteins to perform phagotrophy; i.e., phagolysosome biogenesis, membrane trafficking and the actin cytoskeleton^42^ (Supplementary Data 4). A PCoA showed that *Paraphelidium* and *Rozella* are much closer to one another in terms of phagotrophy- than metabolism-related genes, grouping with fungi and far from Microsporidia (Fig. 3c and Supplementary Fig. 4a). This pattern was also evident in the presence/absence matrix (Fig. 3d and Supplementary Fig. 4b). In both, the two species clustered with early-branching fungi: chytridiomycetes (*Spizellomyces*, *Batrachochytrium*, *Gonapodya*) and blastocladiomycetes (*Allomyces*, *Catenaria*, *Blastocladiella*). The clustering of *Rozella* and *Paraphelidium* with fungi based on 11 phagotrophy-related KOs (695 orthologs) might seem puzzling, since fungi are osmotrophs. However, this clustering is not unexpected, since their phagotrophic function likely involves many genes also involved in other cellular processes (e.g., cytoskeletal movement, endocytosis) that they share with fungi. Only a subset of genes in *Rozella* and *Paraphelidium* (less than 100 genes), seem to be specific and slightly different between *Rozella* and the aphelid. These genes are those likely responsible, together with the genes that they share with fungi, for the phagotrophic function. Furthermore, since *Paraphelidium* and *Rozella* have similar, but not identical, protein sets involved in phagosome, lysosome and endocytic processes, different protein subsets specialized in each lineage for phagocytosis function from their ancestral phagocytic ancestor (Supplementary Fig. 4c–e).

In order to gain more insights into the diversification of the actin cytoskeleton toolkit of fungi and opisthosporidians, we analyzed the evolution of myosin motor proteins. The myosin toolkit of *Paraphelidium* contains a mixture of classical fungal families and others previously identified in holozoans (animals and their unicellular relatives; Supplementary Data 5). We recovered diversified class I myosins in *Paraphelidium*, *Spizellomyces* and nucleariids (Supplementary Fig. 4g), with paralogs of Ic/h and Ik families, previously described only in holozoans^43^. *Paraphelidium*, nucleariids and *Gonapodya* also possess homologs of class XV myosins, formerly thought holozoan-specific^43^. In addition, the aphelid not only possesses homologs of the V/VII myosin-motor family associated to chitin-synthase (see above; Supplementary Fig. 2a), but also myosins of If (pan-eukaryotic), II (amorphean), and XXII (opisthokont) classes, which clustered with previously described fungal homologs (Supplementary Data 5; Supplementary Fig. 4f). Thus, compared with the ancestral opisthokont myosin complement^43^, *Paraphelidium* retains all but one myosin class (VI), with homologs of all myosin families present in fungi (If, II, V, XVII - chitin synthase) plus four additional families (Ic/h, Ik, XV and XXII) that were lost in eumycotan fungi (i.e., fungi to the exclusion of chytrids). This suggests an independent step-wise simplification of the myosin complement in fungi, *Rozella* and Microsporidia, with *Paraphelidium*, nucleariids and chytrids retaining ancestral classes.

Recently, it has been proposed that WASP and SCAR/WAVE genes, encoding activators of branched actin assembly proteins^44^ and hence related to the actin cytoskeleton, are essential to build filopodia and for the cell motility called alpha-motility^45^. The aphelid zoospore motility involves filopodia (Supplementary video 1), resembling that of chytrid fungi^45^. Likewise, its transcriptome contains homologs of WASP and SCAR/WAVE proteins (Supplementary Data 6). Interestingly, although *Rozella* also contains WASP and SCAR/WAVE homologs, filopodia have not been described in its zoospores^46–48^. Therefore, either *Rozella* has some form of not-yet-identified filopodia or the presence of WASP and SCAR/WAVE does not necessarily imply the development of filopodial movement^45^. If this is the case, the *Rozella* genes might instead be involved in phagotrophy in this organism.

## Discussion

From an evolutionary perspective, the current situation in the holomycotan branch (including Fungi) of the eukaryotic super-group Opisthokonta mirrors that of the Holozoa (including Metazoa), where the discovery of deeply-branching unicellular protists that possess genes thought unique to animals continues to challenge previous evolutionary schemes about the emergence of Metazoa^24,49^. Thus, the discovery that a highly diverse set of environmental sequences formed a deeply-branching clade that appeared as sister group to fungi and that included the parasite *Rozella allomyces* (named rozellids^4^, and subsequently Cryptomycota^5^, Rozellomycota^50^, or Rozellosporidia^51^, triggered the discussion of what fungi actually are^1,52^. This debate was further nourished by the discovery that aphelids, another highly diverse group of parasites of algae^10^, formed a large clade with rozellids and Microsporidia based on rRNA and RNA-polymerase genes^7,12,51,53^. This seemingly monophyletic group was named Opisthosporidia and branched as sister group to classical fungi (the clade including from chytrids and their relatives to the Dikarya^52^) in phylogenetic trees^7^. Lately, many mycologists include the three opisthosporidian lineages, Aphelida, Rozellosporidia and Microsporidia, within Fungi^1,9,16,54^. Some authors even incorporate as fungi the free-living phagotrophic chitin-lacking nucleariids, thus pushing the limits of fungi to incorporate all holomycotan (possibly a confusing name) lineages, despite asserting that the kingdom Fungi is characterized by osmotrophic nutrition across a chitinous cell wall^8^. However, unlike fungi, aphelids and rozellids (the deepest branches in the holomycotan clade to the exclusion of nucleariids) are phagotrophs, lack a chitin cell wall at the vegetative stage and are endobiotic with a unique mode of penetration into their hosts^7,11,52,53^. Also, because *Rozella* has a reduced genome, a typical trait of streamlined parasites, some authors inferred a parasitic nature for the ancestor of fungi^16^.

**Fig. 4:**
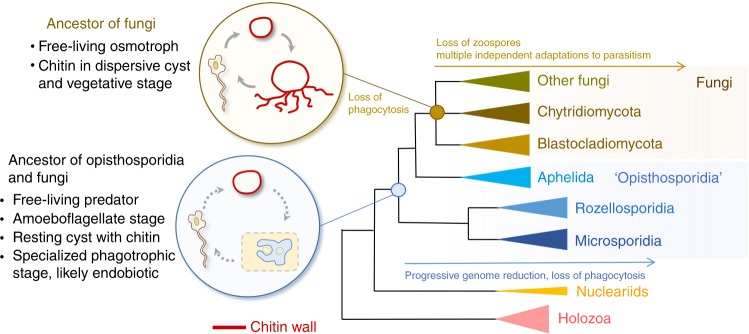
Early evolution of fungi and related lineages. Schematic representation of evolutionary relationships between fungi, rozellids and Microsporidia, within the holomycotan branch of Opisthokonts. Inferred key ancestral features and life cycle stages are depicted at the corresponding ancestral nodes

The study of the *Paraphelidium tribonemae* transcriptome clarifies some of this controversy. Our multi-gene phylogenetic analyses place the aphelids as a pivotal group branching very deeply in the Opisthosporidia/Fungi clade. However, contrary to previous phylogenetic analyses based on only a few genes, Opisthosporidia do not seem to be monophyletic. Aphelids strongly emerge as the sister group to fungi to the exclusion of rozellids and Microsporidia (Fig. 1). Because *Rozella* and aphelids now appear as paraphyletic lineages that share similar ancestral characteristics (e.g., alternate life cycle of zoospores, cyst, phagotrophic ameboid vegetative stages), our results parsimoniously suggest that fungi evolved from ancestors that were similar to aphelids. Because of its deep pivotal position, the comparative study of the *Paraphelidium* transcriptome allows better inference of ancestral states for fungi and the now paraphyletic Opisthosporidia clade (Fig. 4). *Paraphelidium* has a complex metabolism resembling that of free-living chytrids but feeds by phagotrophy like free-living nucleariids and holozoan protists (Fig. 3). This suggests that aphelids are ‘borderline’ parasites (or parasitoids, according to some definitions) that have not undergone the reduction process that characterizes *Rozella* and all members along the microsporidian branch. Being more gene-rich and close to the root separating fungi-aphelids from rozellids-microsporidia, aphelids may have retained more ancestral features^13,15,16^. These features support a free-living opisthosporidian ancestor that had a complex life cycle including chitin-containing resting cysts, amoeboflagellate zoospores and a phagotrophic amoeba stage possibly specialized in endobiotic predation (Fig. 4). By contrast, the fungal ancestor was a free-living osmotroph that had amoeboflagellate zoospores and chitin in the vegetative stage. From their aphelid-like opisthosporidian free-living ancestor, the fungal lineage lost phagotrophy, acquiring its ecologically successful osmotrophy whereas, on the rozellid line, endobiotic phagotrophic predation shifted into obligate parasitism with more complex life cycles, highly specialized morphologies and genome and metabolic reduction. The analysis of additional genomes/transcriptomes for other aphelids, rozellids and deep-branching fungi should help establishing a solid phylogenomic framework to validate and refine this evolutionary scenario.

## Methods

### Cultures

*Paraphelidium tribonemae* was maintained in an enriched culture with its host *Tribonema gayanum* in mineral medium or in Volvic™ at room temperature in the presence of white light^11^. The culture was progressively cleaned from other heterotrophic eukaryotes by micromanipulation and transfer of infected host filaments to uninfected *T. gayanum* cultures.

### WGA staining, epifluorescence, and scanning electron microscopy

To detect chitin in different cell cycle stages of *Paraphelidium tribonemae*, we incubated actively growing cultures with 5 µg/mL WGA conjugated to Texas Red (Life Technologies) for 10 min at room temperature. After rinsing with Volvic™ water, cells were then observed under a LEICA DM2000LED fluorescence microscope with an HCX_PL_FLUOTAR 100×/1.30 oil PH3 objective. Pictures were taken with a LEICA DFC3000G camera using the LEICA Application Suite v4.5 and edited with ImageJ (http://imagej.nih.gov/ij/). For SEM observations, we transferred 1-week-old cultures to a Petri dish containing mineral medium and two coverslips. Cells were let to settle overnight before fixation (1% OsO_4_ and 1% HgCl_2_ for 45 min). After washing (3 × 10 min in distilled water), samples were dehydrated in ethanol series (30, 50, 70, 90, 96, and 100%) for 10 min each. After critical-point drying and sputter-coating with platinum, cells were visualized with a Zeiss Sigma FE-SEM at 1 kV acceleration voltage.

### Transcriptome sequencing

To obtain a complete representation of the aphelid transcriptome across different cell-cycle stages, we extracted RNA from a young culture (3 days after inoculation) rich in aphelid zoospores and cysts, and eight old cultures (5–7 days after inoculation) with few remaining *T. gayanum* living cells and rich in aphelid plasmodia, resting spores and zoospores. To minimize algal overrepresentation, we maintained the cultures in the dark. Total RNA was extracted with the RNeasy mini Kit (Qiagen), quantified by Qubit (ThermoFisher Scientific) and sent to Eurofins Genomics (Germany) for *de novo* transcriptome sequencing. Two cDNA Illumina libraries for, respectively, the young and old enrichment cultures were constructed after polyA mRNA selection and paired-end (2 × 125 bp) sequenced with Illumina HiSeq 2500 Chemistry v4.

### Transcriptome assembly, decontamination and annotation

A total of 71,015,565 and 58,826,379 reads, respectively, were obtained for the libraries of young and old cultures. From these, we obtained 34,844,871 (young) and 25,569,845 (old) paired-end reads. After quality/Illumina Hiseq adapter trimming using Trimmomatic v0.33^55^, we retained 8,601,196 forward and 6,535,164 reverse reads (young) and 10,063,604 forward and 4,563,537 reverse unpaired reads (old). Resulting reads were assembled using Trinity^56^ with min_kmer_cov 2 and normalize_reads options. This initial assembly, Partr_v0, contained 68,130 contigs. 47 of them were identified as ribosomal RNAs using RNAMMER^57^ and included sequences of *Paraphelidium tribonemae*, its host *Tribonema gayanum*, chloroplasts and bacteria. No other eukaryotic ribosomal genes were detected. To decontaminate *P. tribonemae* from host and bacterial sequences, we used Blobtools^58^ and, as input files, our assembly, a read map file obtained with the fast gapped-read alignment program Bowtie2^59^ and a formatted hit list obtained after applying diamond-blastx^60^ to our sequences against the NCBI RefSeq database (*e*-value threshold 1e−10). To build the blobDB file, we used the first 10 hits for each putative transcript with the bestsum as tax-rule criterion to get a taxonomic affiliation at species rank. Sequences with no hit (41,836 sequences, 61.4% of the assembly) and sequences affiliated to bacteria, archaea, or viruses were removed. The remaining 21,688 eukaryotic sequences were blasted against a partial REFSEQ protein database (*e*-value threshold 1e−10) containing sequences of 5 fungi (*Spizellomyces punctatus* DAOM BR117, *Batrachochytrium dendrobatidis* JAM81, *Agaricus bisporus* var. *bisporus* H97, *Cryptococcus neoformans* var. *grubii* H99 and *Cryptococcus neoformans* var. *neoformans* JEC21) and 6 stramenopiles (*Nannochloropsis gaditana* CCMP526, *Phytophthora sojae, Phytophthora parasitica* INRA-310, *Phytophthora infestans* T30–4, *Aureococcus anophagefferens* and *Aphanomyces invadans*). If the first hit was a stramenopile (potential host origin), the sequence was discarded. 13,786 transcripts appeared free of contamination. Transdecoder v2 (http://transdecoder.github.io) with—search_pfam option yielded 16,841 peptide sequences that were filtered using Cd-hit v4.6^61^ with a 100% identity resulting in 13,035 peptides. These peptides were functionally annotated with eggNOG-mapper^38^ using both DIAMOND and HMMER mapping mode, eukaryotes as taxonomic scope, all orthologs and non-electronic terms for Gene Ontology evidence. This resulted in 10,669 annotated peptides for the *Paraphelidium tribonemae* predicted proteome (version Partr_v1). Finally, we generated transcriptomic data for the *Tribonema gayanum* host using the same methods and parameters for sequencing, assembly and annotation, as described above. After blasting Partr_v1 against a database of *Tribonema gayanum* proteins, we further excluded 219 + 11 additional proteins that were 100% or >95% identical to *Tribonema* proteins. After these additional cleaning steps, version 1.5 of the *Paraphelidium tribonemae* predicted proteome contained 10,439 proteins (Supplementary Data 1). The completeness of this new transcriptome was estimated to 91.4% with BUSCO^17^ using the eukaryotic ortholog database 9 (protein option).

### Phylogenomic analyses

We used three different protein datasets previously used for phylogenomic analyses of Opisthokonta and, more specifically, Microsporidia that included, respectively, 93 single-copy protein domains (SCPD)^18^, 53 proteins (BMC)^19^ and 259 proteins (GBE)^15^. We updated these datasets with sequences from the *Paraphelidium tribonemae* transcriptome, thirteen derived Microsporidia as well as *Mitosporidium daphniae*^20^, *Paramicrosporidium saccamoebae*^21^ and *Amphiamblys* sp^15^. Some stramenopile taxa such *Ectocarpus siliculosus*^62^, *Thalassiosira pseudonana*^63^ or *Phytophthora infestans* (GenBank NW_003303758.1) were also included to further prevent any previously undetected *Tribonema gayanum* contamination. Markers were aligned with MAFFT v7^64^, using the method L-INS-i with 1000 iterations. Unambiguously aligned regions were trimmed with TrimAl^65^ with the automated1 algorithm, then approximate ML trees were inferred using FastTree^66^, all visually inspected in Geneious^67^ to check for possible contamination. Contamination-free alignments were relieved from distant outgroup taxa. For in-depth analyses, we used opisthokont, mainly holomycotan, sequences, including apusomonad and breviate sequences as outgroup. We then made phylogenomic analyses including or excluding thirteen fast-evolving microsporidian parasites (49 or 36 species). Sequences of the selected taxa were concatenated using Alvert.py from the package Barrel-o-Monkeys (http://rogerlab.biochemistryandmolecularbiology.dal.ca/Software/Software.htm#Monkeybarrel). This resulted in alignments containing the following number of amino acids as a function of dataset and taxon sampling: 22,976 (SCPD49); 15,744 (BMC49); 84,810 (GBE49); 24,413 (SCPD36); 16,840 (BMC36); 96,640 (GBE36). Bayesian phylogenetic trees were inferred using PhyloBayes-MPI v1.5^68^ under the CAT-Poisson evolutionary model, applying the Dirichlet process and removing constant sites. Two independent MCMC chains for each dataset were run for >15,000 generations, saving one every 10 trees. Phylogenetic analyses were stopped once convergence thresholds were reached after a burn-in of 25% (i.e., maximum discrepancy < 0.1 and minimum effective size > 100 using bpcomp). We also applied ML phylogenetic reconstruction using IQ-TREE^69^ with the profile mixture model C60^23^. Statistical support was obtained with 1000 ultrafast bootstraps^70^ and 1000 replicates of the SH-­like approximate likelihood ratio test^71^. To alleviate the local computational burden, many of these analyses were carried out using CIPRES Science Gateway^72^. Trees were visualized with FigTree (http://tree.bio.ed.ac.uk/software/figtree/). To test if the best topology obtained was significantly better than other alternatives (Supplementary Data 2), we tested whether constrained alternative topologies could be rejected for different datasets. We used Mesquite^73^ to constrain topologies representing the opisthosporidian monophyly (aphelids + rozellids + microsporidians) and the monophyly of aphelids with either blastocladiomycetes, chytridiomycetes or chytridiomycetes + blastocladiomycetes. The constrained topologies without any branch-length were reanalyzed with the -g option of IQ-TREE and the best-fitting model for each dataset. The resulting trees were then concatenated and AU tests were performed for each dataset with the -z and -au options as described in the advanced documentation of IQ-TREE. To minimize systematic error due to the inclusion of fast-evolving sites in our protein datasets, we progressively removed the fastest evolving sites at steps of 5% sites removed at a time. Among-site evolutionary rates were inferred using IQ-TREE -wsr option and its best-fitting model for each dataset (Supplementary Data 2). A total of 19 subsets were created for each dataset. We then reconstructed phylogenetic trees using IQ-TREE with the same best fitting model as for the whole dataset. To know how supported were alternative topologies in bootstrapped trees, we used CONSENSE from the PHYLIP package^74^ and interrogated the.UFBOOT file using a Python script (M Kolisko, pers. comm).

### Homology searches and phylogenetic analyses of specific proteins

Protein sets used in this study were obtained in May 2017 from the NCBI protein database (https://www.ncbi.nlm.nih.gov/protein/), except the following: *Chromosphaera perkinsii*, *Corallochytrium limacisporum* and *Ichthyophonus hoferi* genome data, obtained from the ref. ^24^; *Spizellomyces punctatus, Gonapodya prolifera, Batrachochytrium dendrobatidis, Allomyces macrogynus, Catenaria anguillulae* and *Blastocladiella britannica*, retrieved from MycoCosm portal, Joint Genome Institute* (http://www.jgi.doe.gov/); *Parvularia atlantis* (previously *Nuclearia* sp. ATCC50694, from 10.6084/m9.figshare.3898485.v4); and *Paramicrosporidium saccamoebae*, from NCBI in January 2018. [* These sequence data were produced by the US Department of Energy Joint Genome Institute http://www.jgi.doe.gov/ in collaboration with the user community]. Query sequences to retrieve chitin and related cell-wall proteins (chitin synthases (CHS), chitin deacetylases (CDA), and chitinases (CTS)) were obtained from the study of *Rozella allomycis*^16^ except for 1,3-beta glucan synthase (FKS1), which was obtained from *Saccharomyces cerevisiae* S288C. We used sequences from *Aspergillus fumigatus* as initial queries for cellulases^34^ and also opisthokont homologs retrieved by BLASTp against the *Paraphelidium* transcriptome. Homology was confirmed by searching for the identified sequences in the eggnog^38^ annotation file (Supplementary Data 1), performing reverse BLAST against the non-redundant NCBI database and HMMER analyses^75^. Cell-wall proteins, notably chitinases and cellulases, were also annotated using CAZyme identification tools (http://csbl.bmb.uga.edu/dbCAN/annotate.php). We produced comprehensive alignments including sequences retrieved from previous studies and sequences identified by BLAST in GenBank or in the mycoCLAP database^35^ with the MAFFT web server^64^ applying the progressive L-INS-i algorithm (except for the multidomain CHS sequences for which E-INS-i was used). Alignments were trimmed from gaps and ambiguously aligned sites using Trimal^65^ with the automated1 algorithm. ML trees were inferred using IQ-TREE web server^69^ with the LG + G4 evolutionary model and visualized with FigTree. In the case of myosins, protein sequences were retrieved by querying the HMM profiles of the myosin head domain (Pfam accession: PF00063) against our database of *Paraphelidium tribonemae* proteins, using the *hmmersearch* utility of HMMER (http://hmmer.org) and the profile-specific gathering threshold cut-off. For each retrieved homolog, the protein segment spanning the Pfam-defined myosin head domain was aligned against a pan-eukaryotic alignment of myosins^43^, using MAFFT^64^ with the G-INS-i algorithm optimized for global sequence homology. The alignment was run for up to 10³ cycles of iterative refinement and manually examined, curated and trimmed. The ML phylogenetic reconstruction of the trimmed alignment (997 sequences, 400 sites) was done with IQ-tree^69^ under the LG model with a 10-categories free-rate distribution, selected as the best-fitting model according to the IQ-TREE *TESTNEW* algorithm as per the Bayesian information criterion. The best-scoring tree was searched for up to 100 iterations, starting from 100 initial parsimonious trees; statistical supports for the bipartitions were drawn from 1000 ultra-fast bootstrap^70^ replicates with a 0.99 minimum correlation as convergence criterion, and 1000 replicates of the SH-like approximate likelihood ratio test. We classified the myosin homologs of *Paraphelidium tribonemae* according to the phylogenetic framework of Sebé-Pedrós et al^43^. To characterize the classified homologs (Supplementary Data 5), we recorded the protein domain architectures of the full-length proteins using Pfamscan and the 29th release of the Pfam database^76^.

### Comparative analyses of proteins involved in primary metabolism, cytoskeleton, membrane-trafficking, and phagotrophy

To get a broad view of the metabolic capabilities of *Paraphelidium tribonemae* in comparison with parasitic and free-living organisms, we performed a statistical multivariate analysis similar to that previously used for a similar purpose in *Rozella allomycis*^16^. We initially searched in *P. tribonemae* for the presence of 6469 unique orthologous groups as per eggnog^38^, corresponding to 8 primary metabolism categories (Gene Ontology, GO). The correspondence between GO terms and primary metabolism COGs was obtained from http://geneontology.org/external2go/cog2go, and was as follows: [C] Energy production and conversion (912 orthologs); [G] Carbohydrate transport and metabolism (1901 orthologs); [E] Amino acid transport and metabolism (714 orthologs); [F] Nucleotide transport and metabolism (351 orthologs); [H] Coenzyme transport and metabolism (375 orthologs); [I] Lipid transport and metabolism (965 orthologs); [P] Inorganic ion transport and metabolism (842 orthologs) and [Q] Secondary metabolites biosynthesis, transport and catabolism (570 orthologs). From these categories, we identified 1172 non-ambiguous orthologs in the *P. tribonemae* transcriptome that were shared with a set of other 41 protist species (Supplementary Data 3). These 41 protist species represent a broader taxonomic sampling as compared to the *R. allomyces* study^16^. We included 11 opisthosporidians: *Paramicrosporidium saccamoebae* (Par_sa), *Amphiamblys* sp. (Amp_sp), *Antonospora locustae* (Ant_lo), *Encephalitozoon cuniculi* (Enc_cu), *Encephalitozoon intestinalis* (Enc_in), *Enterocytozoon bieneusi* (Ent_bi), *Mitosporidium daphniae* (Mit_da), *Nematocida parisii* (Nem_pa), *Nosema ceranae* (Nos_ce), *Rozella allomycis* (Roz_al), *Paraphelidium tribonemae* (Par_tr); 10 fungi covering most fungal phyla: *Spizellomyces punctatus* (Spi_pu), *Gonapodya prolifera* (Gon_pr), *Batrachochytrium dendrobatidis* (Bat_de), *Allomyces macrogynus* (All_ma), *Catenaria anguillulae* (Cat_an), *Blastocladiella britannica* (Bla_br), *Mortierella verticillata* (Mor_ve), *Coprinopsis cinerea* (Cop_ci), *Ustilago maydis* (Ust_ma), *Schizosaccharomyces pombe* (Sch_po); 2 nucleariids *Fonticula alba* (Fon_al), *Parvularia atlantis* (Par_at); 7 holozoans: *Salpingoeca rosetta* (Sal_ro), *Monosiga brevicollis* (Mon_br), *Capsaspora owczarzaki* (Cap_ow), *Chromosphaera perkinsii* (Chr_pe), *Corallochytrium limacisporum* (Cor_li), *Ichthyophonus hoferi* (Ich_ho), *Sphaeroforma arctica* (Sph_ar); 3 amoebozoans: *Acanthamoeba castellanii* (Aca_ca), *Dictyostelium purpureum* (Dic_pu), *Polysphondylium pallidum* (Pol_pa), *Entamoeba histolytica* (Ent_hi); the free-living apusomonad *Thecamonas trahens* (The_tr), *Naegleria gruberi* (Nae_gr) and 6 other eucaryotic parasites: *Toxoplasma gondii* (Tox_go), *Plasmodium falciparum* (Pla_fa), *Trypanosoma brucei* (Try_br), *Leishmania major* (Lei_ma), *Phytophora infestans* (Phy_in). We annotated all 41 protein sets using eggNOG-mapper^38^ using DIAMOND mapping mode, eukaryotes as taxonomic scope, all orthologs and non-electronic terms for Gene Ontology evidence. Species-wise ortholog counts were transformed into a presence/absence matrix (encoded as 0/1), or binary ortholog profile. This binary ortholog profile excluded all orthologs with no identifiable orthologs in any of the 41 species used in this analysis (e.g., proteins related to exclusive prokaryotic metabolism or photosynthesis) leaving a total number of 1172 primary metabolism ortholog (Supplementary Data 3). The similarity between each species’ binary COG profile was assessed using the Pearson’s correlation coefficient (*r* statistic) as implemented in the R *stats* library^77^. We built a complementary species distance matrix by defining dissimilarity as 1-*r*., and we analyzed this dissimilarity matrix using a Principal Coordinate Analysis (PCoA) as implemented in the R *ape* library^78^, with default parameters. For each PCoA, we represented the two vectors with the highest fraction of explained variance, which were in all cases higher than the fractions expected under the broken stick model. In addition, we plotted binary OG profiles of each species in a presence/absence heatmap, produced using the heatmap.2 function in the R *gplots* library^79^. Species’ order was defined by a Ward hierarchical clustering of the aforementioned interspecific Pearson’s correlation coefficients. Ortholog order was defined by Ward hierarchical clustering on euclidean distances. All clustering and distance analyses were performed using R *stats* library^77^. We represented the raw species clustering (Pearson correlation + Ward) in a separate heatmap, scaling the color code to display positive Pearson correlation values (0–1). Finally, each sub-set of primary metabolism COGs was also analyzed separately (categories C, E, F, G, H, I, P, and Q) using the same hierarchical clustering as above to cluster species according to their metabolism gene content (based on Pearson + Ward). We carried out similar comparisons and statistical analyses for proteins involved in phagotrophy, membrane trafficking and cytoskeleton. Briefly, we looked for proteins broadly related to those systems (actin cytoskeleton, exocytosis, fungal vacuole, exosome, endosome, mitochondrial biogenesis, ER-Golgi, lysosome, autophagy, peroxisome) in the KEGG BRITE reference database (http://www.genome.jp/kegg/brite.html). We identified 849 proteins related to membrane trafficking (ko04131), 279 cytoskeletal proteins (ko04812) and 361 proteins related to phagotrophy (KEGG map04144_endocytosis, map04145_phagosome, map04146_peroxisome, map04142_lysosome, map04810_actin_regulation, map04141_autophagy and map04071_sphingolipid_signal), which were used to carry out PCoA and clustering analyses as above (Supplementary Fig. 4). Since the eggNOG annotation file did not provide specific KEGG orthologs but only KEGG maps, we used the following approach to get the KEGG ortholog profile for the 41 taxa. All proteins in 35 eukaryote genomes^80^ were clustered to a non-redundant set using cd-hit at a 90% identity threshold. Those 35 nr90 genomes were compared in an all-vs-all BLAST analysis with an e-value cutoff of 1 × 10^−3^. The all-vs-all BLAST output was clustered using the MCL algorithm with an inflation parameter of 2.0. To avoid lineage specific proteins, the resultant clusters were retained if the proteins in the cluster were derived from 3 or more organisms. Proteins from each cluster were aligned using MAFFT, the alignments trimmed with trimAl, and HMM profiles built for each alignment using HMMer3. This analysis resulted in a set of 14,095 HMMs. All proteins from the UniProt-SwissProt database were searched against the entire set of 14,095 HMMs. Each HMM was assigned a best-hit protein annotation from the UniProt-SwissProt database. KEGG ortholog identifiers (IDs) associated with each HMM were obtained from UniProtKB IDs and KEGG ortholog IDs using the mappings available from UniProtKB. Additional KEGG ortholog IDs were mapped to the HMMs by searching the HMMs against all proteins annotated to a given KEGG ortholog ID. The best hit bit score among those proteins to the HMM was compared to the best-hit bit score of the UniProtKB annotation for that HMM. If the best hit KEGG ortholog ID bit score was at least 80% of the best hit UniProtKB protein bit score, and there was no KEGG ortholog ID already associated with the UniProtKB annotation, the KEGG ortholog ID was transferred to that HMM. To build a presence-absence matrix associated with KEGG ortholog IDs, the HMMs were searched against all proteins in a genome. Best hit HMMs below threshold (total protein *e*-value ≤ 1 × 10^−5^ and a best single domain *e*-value ≤ 1e−4) were assigned for each protein in a genome. The best hit HMMs and their associated KEGG ortholog IDs were used for presence/absence calls. If at least one protein in a genome had a best hit to a given HMM, the associated KEGG ortholog was considered present for that genome. From a total of 1568 orthologs associated with the KEGG maps of interest, 633 KEGG ortholog IDs were identified in the UniProtKB annotations of the HMMs. An additional 62 KEGG ortholog IDs were mapped to HMMs by considering similarity of bit scores between the best UniProt/SwissProt protein hit to the HMM and the best KEGG ortholog ID protein hit to the HMM (Supplementary Data 3). Those 695 KEGG ortholog IDs were used to perform the same multivariate statistical analyses as per the metabolism section for the 41 taxa. The phagolysosome KEGG maps in *Paraphelidium tribonemae* and *Rozella allomycis* were compared using KEGG tools. To do so, we first annotated the two protein sets using BlastKOALA^81^ with eukaryotes as taxonomy group and genus_eukaryotes KEGG GENES database. We then uploaded our annotations in the KEGG Mapper Reconstruct Pathway and BRITE servers.

### Code availability

Custom scripts have been deposited in Github (https://github.com/xgrau/paraphelidium2018).

## Electronic supplementary material

Supplementary Information

Description of Additional Supplementary Files

Supplementary Data 1

Supplementary Data 2

Supplementary Data 3

Supplementary Data 4

Supplementary Data 5

Supplementary Data 6

Supplementary Software 1

Supplementary Movie 1

## Data Availability

Raw read sequences have been deposited in NCBI under accession number PRJNA402032. *Paraphelidium tribonemae* metatranscriptomic nucleotide contig assembly and version 1.5 of the predicted proteome are deposited in figshare^82,83^ under Creative Commons 4.0 licence. All trees are available as Supplementary file all_trees.txt.

## References

[1] Richards, T. A., Leonard, G. & Wideman, J. G. What defines the “Kingdom” fungi? In Heitman et al. (ed.), *The fungal Kingdom*. p. 57–77 (ASM Press, Washington, 2017). 10.1128/microbiolspec.funk-0044-2017 PMC11687502 28643626

[2] James TY (2006). Reconstructing the early evolution of Fungi using a six-gene phylogeny. Nature.

[3] James TY (2006). A molecular phylogeny of the flagellated fungi (Chytridiomycota) and description of a new phylum (Blastocladiomycota). Mycologia.

[4] Lara E, Moreira D, Lopez-Garcia P (2010). The environmental clade LKM11 and Rozella form the deepest branching clade of fungi. Protist.

[5] Jones MD (2011). Discovery of novel intermediate forms redefines the fungal tree of life. Nature.

[6] Bass, D. et al. Clarifying the relationships between microsporidia and cryptomycota. *J. Eukaryot. Microbiol.* 10.1111/jeu.12519 (2018). 10.1111/jeu.12519 PMC6282948 29603494

[7] Karpov SA (2014). Morphology, phylogeny, and ecology of the aphelids (Aphelidea, Opisthokonta) and proposal for the new superphylum Opisthosporidia. Front. Microbiol..

[8] Berbee ML, James TY, Strullu-Derrien C (2017). Early diverging fungi: diversity and impact at the dawn of Terrestrial life. Annu. Rev. Microbiol..

[9] Spatafora, J. W. et al. The fungal tree of life: from molecular systematics to genome-scale phylogenies. In Heitman et al. (ed.), *The fungal Kingdom*. p. 3–34 (ASM Press, Washington, DC, 2017). 10.1128/microbiolspec.funk-0053-2016 PMC11687545 28917057

[10] Gromov BV (2000). Algal parasites of the genera *Aphelidium, Amoeboaphelidium*, and *Pseudaphelidium* from the Cienkovski’s “monadinea” group as representatives of a new class. Zool. Zh..

[11] Karpov SA (2017). Morphological and genetic diversity of Opisthosporidia: new aphelid *Paraphelidium tribonemae* gen. et sp. nov. J. Eukaryot. Microbiol..

[12] Karpov SA (2013). Obligately phagotrophic aphelids turned out to branch with the earliest-diverging fungi. Protist.

[13] Corradi N (2015). Microsporidia: eukaryotic intracellular parasites shaped by gene loss and horizontal gene transfers. Ann. Rev. Microbiol..

[14] Letcher PM (2013). Characterization of *Amoeboaphelidium protococcarum*, an algal parasite new to the Cryptomycota isolated from an outdoor algal pond used for the production of biofuel. PLoS One.

[15] Mikhailov KV, Simdyanov TG, Aleoshin VV (2017). Genomic survey of a hyperparasitic microsporidian *Amphiamblys* sp. (Metchnikovellidae). Genome Biol. Evol..

[16] James TY (2013). Shared signatures of parasitism and phylogenomics unite Cryptomycota and Microsporidia. Curr. Biol..

[17] Simao FA, Waterhouse RM, Ioannidis P, Kriventseva EV, Zdobnov EM (2015). BUSCO: assessing genome assembly and annotation completeness with single-copy orthologs. Bioinformatics.

[18] Torruella G (2015). Phylogenomics reveals convergent evolution of lifestyles in close relatives of animals and fungi. Curr. Biol..

[19] Capella-Gutierrez S, Marcet-Houben M, Gabaldon T (2012). Phylogenomics supports microsporidia as the earliest diverging clade of sequenced fungi. BMC Biol..

[20] Haag KL (2014). Evolution of a morphological novelty occurred before genome compaction in a lineage of extreme parasites. Proc. Natl Acad. Sci. USA.

[21] Quandt, C. A. et al. The genome of an intranuclear parasite, *Paramicrosporidium saccamoebae*, reveals alternative adaptations to obligate intracellular parasitism. *Elife* **6**, e29594 (2017). 10.7554/eLife.29594 PMC5701793 29171834

[22] Lartillot N, Philippe H (2004). A Bayesian mixture model for across-site heterogeneities in the amino-acid replacement process. Mol. Biol. Evol..

[23] Le SQ, Lartillot N, Gascuel O (2008). Phylogenetic mixture models for proteins. Philos. Trans. R. Soc. Lond. B.

[24] Grau-Bove X (2017). Dynamics of genomic innovation in the unicellular ancestry of animals. eLife.

[25] Ruiz-Herrera J, Ortiz-Castellanos L (2010). Analysis of the phylogenetic relationships and evolution of the cell walls from yeasts and fungi. Fems. Yeast. Res..

[26] James TY, Berbee ML (2012). No jacket required—new fungal lineage defies dress code: recently described zoosporic fungi lack a cell wall during trophic phase. Bioessays.

[27] Merzendorfer H (2011). The cellular basis of chitin synthesis in fungi and insects: common principles and differences. Eur. J. Cell Biol..

[28] Goncalves IR (2016). Genome-wide analyses of chitin synthases identify horizontal gene transfers towards bacteria and allow a robust and unifying classification into fungi. BMC Evol. Biol..

[29] Vavra J, Lukes J (2013). Microsporidia and ‘the art of living together’. Adv. Parasitol..

[30] Zhao Y, Park RD, Muzzarelli RA (2010). Chitin deacetylases: properties and applications. Mar. Drugs.

[31] Viens P, Lacombe-Harvey ME, Brzezinski R (2015). Chitosanases from family 46 of glycoside hydrolases: from proteins to phenotypes. Mar. Drugs.

[32] Udaya Prakash NA (2010). Evolution, homology conservation, and identification of unique sequence signatures in GH19 family chitinases. J. Mol. Evol..

[33] Cleare M, Percival E (1972). Carbohydrates of the fresh water alga *Tribonema aequale*. I. Low molecular weight and polysaccharides. Brit. Phycol. J..

[34] Kubicek CP, Starr TL, Glass NL (2014). Plant cell wall-degrading enzymes and their secretion in plant-pathogenic fungi. Annu. Rev. Phytopathol..

[35] Murphy, C., Powlowski, J., Wu, M., Butler, G. & Tsang, A. Curation of characterized glycoside hydrolases of fungal origin. *Database* **2011**, bar020 (2011). 10.1093/database/bar020 PMC3263737 21622642

[36] McAndrew RP (2013). From soil to structure, a novel dimeric beta-glucosidase belonging to glycoside hydrolase family 3 isolated from compost using metagenomic analysis. J. Biol. Chem..

[37] Aspeborg H (2012). Evolution, substrate specificity and subfamily classification of glycoside hydrolase family 5 (GH5). BMC Evol. Biol..

[38] Huerta-Cepas J (2016). eggNOG 4.5: a hierarchical orthology framework with improved functional annotations for eukaryotic, prokaryotic and viral sequences. Nucleic Acids Res..

[39] Okuda S (2008). KEGG Atlas mapping for global analysis of metabolic pathways. Nucleic Acids Res..

[40] Roshchina VVNew (2016). Trends and perspectives in the evolution of neurotransmitters in microbial, plant, and animal cells. Adv. Exp. Med. Biol..

[41] Yutin N, Wolf MY, Wolf YI, Koonin EV (2009). The origins of phagocytosis and eukaryogenesis. Biol. Direct..

[42] Boulais J (2010). Molecular characterization of the evolution of phagosomes. Mol. Syst. Biol..

[43] Sebé-Pedrós A, Grau-Bové X, Richards TA, Ruiz-Trillo I (2014). Evolution and classification of myosins, a paneukaryotic whole-genome approach. eLife.

[44] Sebé-Pedrós A (2013). Insights into the origin of metazoan filopodia and microvilli. Mol. Biol. Evol..

[45] Fritz-Laylin LK, Lord SJ, Mullins RD (2017). WASP and SCAR are evolutionarily conserved in actin-filled pseudopod-based motility. J. Cell. Biol..

[46] Held AA (1975). Zoospore of *Rozella allomycis*—ultrastructure. Can. J. Bot..

[47] Held AA (1980). Development of rozella in allomyces—a single zoospore produces numerous zoosporangia and resistant sporangia. Can. J. Bot..

[48] Letcher PM (2017). Morphological, molecular, and ultrastructural characterization of *Rozella rhizoclosmatii*, a new species in Cryptomycota. Fungal Biol..

[49] Hehenberger E (2017). Novel predators reshape Holozoan phylogeny and reveal the presence of a two-component signaling system in the ancestor of animals. Curr. Biol..

[50] Corsaro D (2014). Microsporidia-like parasites of amoebae belong to the early fungal lineage Rozellomycota. Parasitol. Res..

[51] Karpov SA, Torruella G, Moreira D, Mamkaeva MA, López-García P (2017). Molecular phylogeny of *Paraphelidium letcheri* sp. nov. (Aphelida, Opisthosporidia). J. Eukaryot. Microbiol..

[52] Cavalier-Smith T (2013). Early evolution of eukaryote feeding modes, cell structural diversity, and classification of the protozoan phyla Loukozoa, Sulcozoa, and Choanozoa. Eur. J. Protistol..

[53] Karpov SA, Mamkaeva MA, Benzerara K, Moreira D, Lopez-Garcia P (2014). Molecular phylogeny and ultrastructure of *Aphelidium* aff. *melosirae* (Aphelida, Opisthosporidia). Protist.

[54] Tedersoo, L. & Lindahl, B. Fungal identification biases in microbiome projects. *Environ. Microbiol. Rep.* **8**, 774–779 (2016). 10.1111/1758-2229.12438 27348848

[55] Bolger AM, Lohse M, Usadel B (2014). Trimmomatic: a flexible trimmer for Illumina sequence data. Bioinformatics.

[56] Grabherr MG (2011). Full-length transcriptome assembly from RNA-Seq data without a reference genome. Nat. Biotechnol..

[57] Lagesen K (2007). RNAmmer: consistent and rapid annotation of ribosomal RNA genes. Nucleic Acids Res..

[58] Laetsch DR, Blaxter ML (2017). BlobTools: Interrogation of genome assemblies [version 1; referees: 2 approved with reservations]. F1000Res..

[59] Langmead B, Salzberg SL (2012). Fast gapped-read alignment with Bowtie 2. Nat. Methods.

[60] Buchfink B, Xie C, Huson DH (2015). Fast and sensitive protein alignment using DIAMOND. Nat. Methods.

[61] Fu L, Niu B, Zhu Z, Wu S, Li W (2012). CD-HIT: accelerated for clustering the next-generation sequencing data. Bioinformatics.

[62] Cock JM (2010). The *Ectocarpus* genome and the independent evolution of multicellularity in brown algae. Nature.

[63] Armbrust EV (2004). The genome of the diatom *Thalassiosira pseudonana*: ecology, evolution, and metabolism. Science.

[64] Katoh K, Kuma K, Toh H, Miyata T (2005). MAFFT version 5: improvement in accuracy of multiple sequence alignment. Nucleic Acids Res..

[65] Capella-Gutiérrez S, Silla-Martinez JM, Gabaldon T (2009). trimAl: a tool for automated alignment trimming in large-scale phylogenetic analyses. Bioinformatics.

[66] Price MN, Dehal PS, Arkin AP (2010). FastTree 2—approximately maximum-likelihood trees for large alignments. PLoS One.

[67] Kearse M (2012). Geneious Basic: an integrated and extendable desktop software platform for the organization and analysis of sequence data. Bioinformatics.

[68] Lartillot N, Lepage T, Blanquart S (2009). PhyloBayes 3: a Bayesian software package for phylogenetic reconstruction and molecular dating. Bioinformatics.

[69] Nguyen LT, Schmidt HA, von Haeseler A, Minh BQ (2015). IQ-TREE: a fast and effective stochastic algorithm for estimating maximum-likelihood phylogenies. Mol. Biol. Evol..

[70] Minh BQ, Nguyen MA, von Haeseler A (2013). Ultrafast approximation for phylogenetic bootstrap. Mol. Biol. Evol..

[71] Anisimova M, Gil M, Dufayard JF, Dessimoz C, Gascuel O (2011). Survey of branch support methods demonstrates accuracy, power, and robustness of fast likelihood-based approximation schemes. Syst. Biol..

[72] Miller, M. A., Pfeiffer, W. & Schwartz, T. Creating the CIPRES Science Gateway for inference of large phylogenetic trees. *Proceedings of the Gateway Computing Environments Workshop (GCE)*, p. 1–8 (New Orleans, LA, 2010).

[73] Mesquite: a modular system for evolutionary analysis. Version3.40 (2018).

[74] PHYLIP - Phylogeny Inference Package v. 3.6 (University of Washington, Seattle, WA, 1999).

[75] Finn RD, Clements J, Eddy SR (2011). HMMER web server: interactive sequence similarity searching. Nucleic Acids Res..

[76] Finn RD (2016). The Pfam protein families database: towards a more sustainable future. Nucleic Acids Res..

[77] R: A language and environment for statistical computing. v. http://www.r-project.org (R Foundation for Statistical Computing, Vienna, Austria, 2017).

[78] Paradis E, Claude J, Strimmer K (2004). APE: analyses of phylogenetics and evolution in R language. Bioinformatics.

[79] Warnes GR (2016). gplots: various R programming tools for plotting data. R. Package Version.

[80] Burns JA, Pittis AA, Kim E (2018). Gene-based predictive models of trophic modes suggest Asgard archaea are not phagocytotic. Nat. Ecol. Evol..

[81] Kanehisa M, Sato Y, Morishima K (2016). BlastKOALA and GhostKOALA: KEGG tools for functional characterization of genome and metagenome sequences. J. Mol. Biol..

[82] Torruella, G. et al. 10.6084/m9.figshare.7339469.v1 (2018).

[83] Torruella, G. & Ruiz-Trillo, I. Transcriptome of *Parvularia atlantis* (Nuclearia sp. ATCC50694) 10.6084/m9.figshare.3898485.v4 (2017).

