# Supplementary material for: Global transcriptome analysis of the aphelid Paraphelidium tribonemae supports the phagotrophic origin of fungi
Source: Commun Biol. 2018 Dec 19;1:231. doi: 10.1038/s42003-018-0235-z (PMC6299283; doi:10.1038/s42003-018-0235-z)
Supplement: Supplementary file 1 — Supplementary Information [file 42003_2018_235_MOESM1_ESM.pdf]

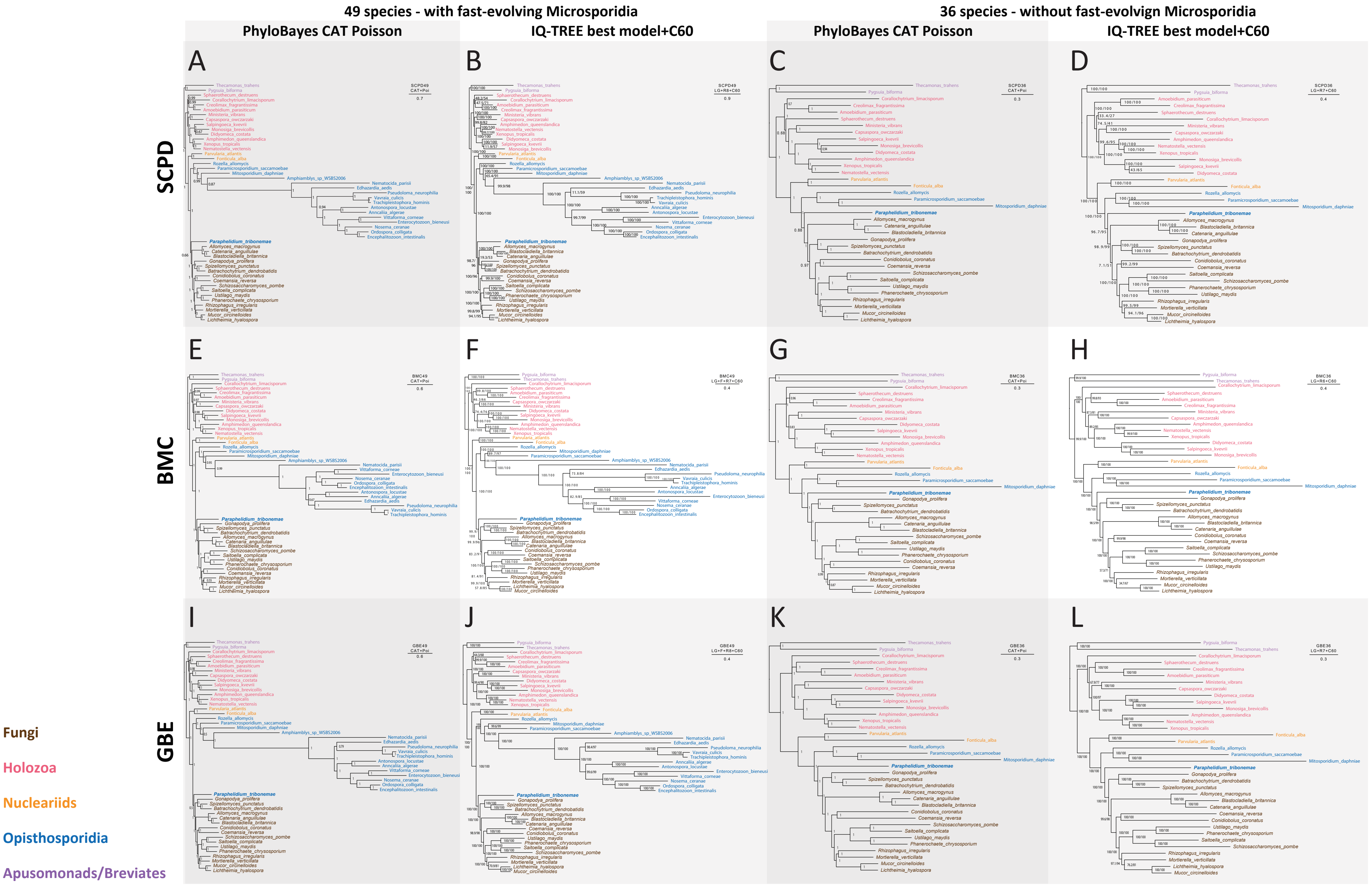

Supplementary Figure 1. Bayesian and Maximum Likelihood (ML) phylogenomic trees showing the position of *Paraphelidium tribonemae*.

Bayesian and ML trees were carried out with three different datasets (SCPD, Torruella et al 2015; BMC, Capella-Gutiérrez et al. 2012; GBE, Mikhailov et al. 2016) for two sets of species including or not long-branch microsporidians (49 species and 36 species, respectively): (C-D) SCPD49; (E-F) SCPD36; (G-H) BMC49 ; (I-J) BMC36; (K-L) GBE49 and (M-N) GBE36. Bayesian phylogenetic trees correspond to the consensus tree from 2 chains of PhyloBayes-MPI using CAT-Poisson mixture model of evolution. Statistical support is in the form of posterior probabilities. ML trees were reconstructed using IQ-TREE with the best fitting model of evolution (indicated in each panel) + C60. Statistical support in ML trees represents SH-aLRT / 1000 ultrafast bootstrap replicates (in %).

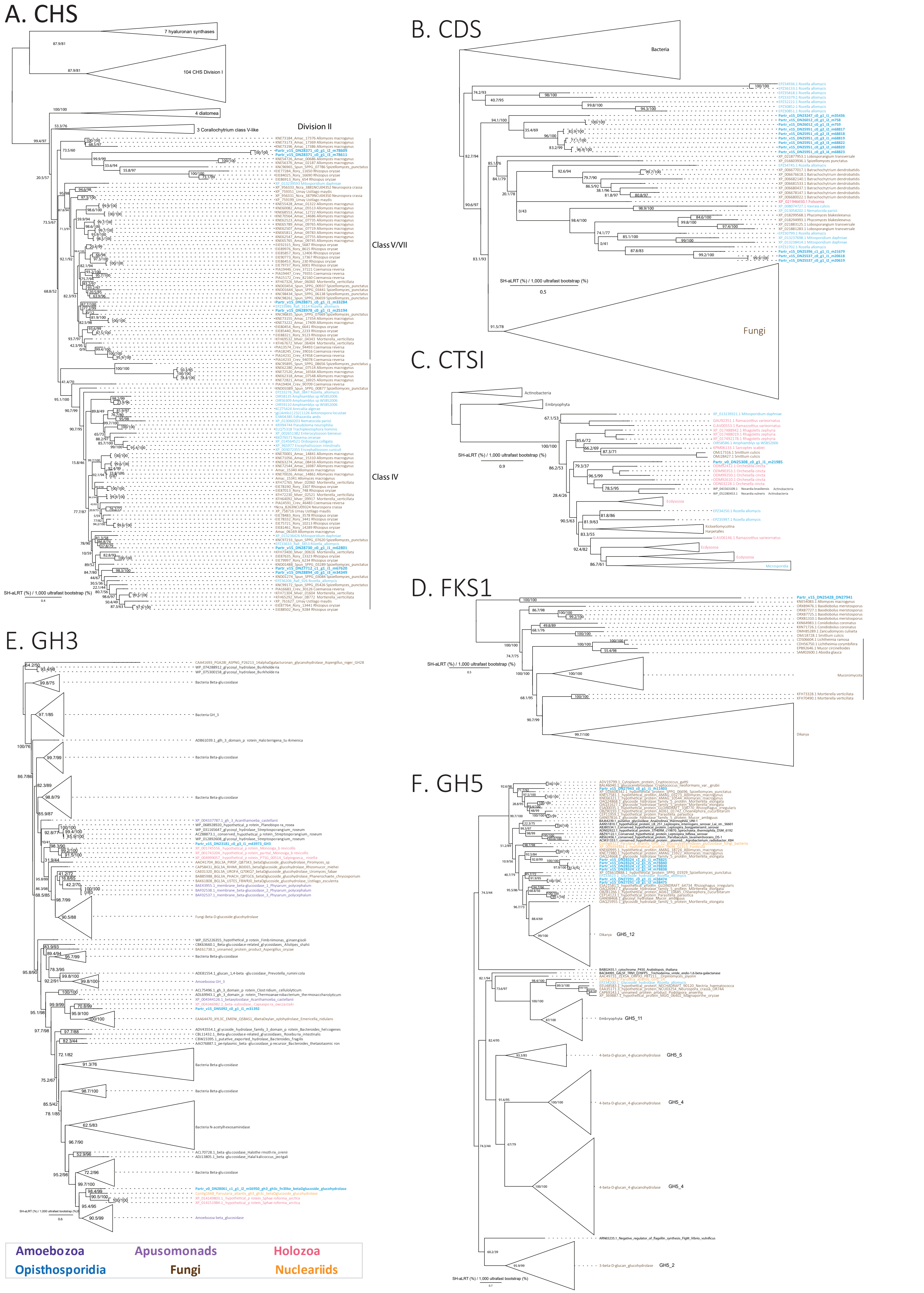

Supplementary Figure 2. Maximum Likelihood phylogenetic trees for cell-wall related proteins (chitin metabolism and cellulases). (A) Chitin synthases (CHS). (B) Chitin deacetylase (CDA). (C) Chitinase class I (CTS I) Glycoside hydrolase 19. (D) 1,»-beta-glucan synthase (FKS1). To build this tree, the two domains PF02364 and PF14288 encoded by separate transcripts in *Paraphelidium* have been concatenated. (E) Glycoside hydrolase family 3 (GH3). (F) Glycoside hydrolase family 5 (GH5). Topologies and statistical supports were obtained with IQTREE LG NEWTEST model of evolution. Node support (in %) represent 1,000 SH-like approximate likelihood ratio test (SH-aLRT) and ultrafast bootstraps.

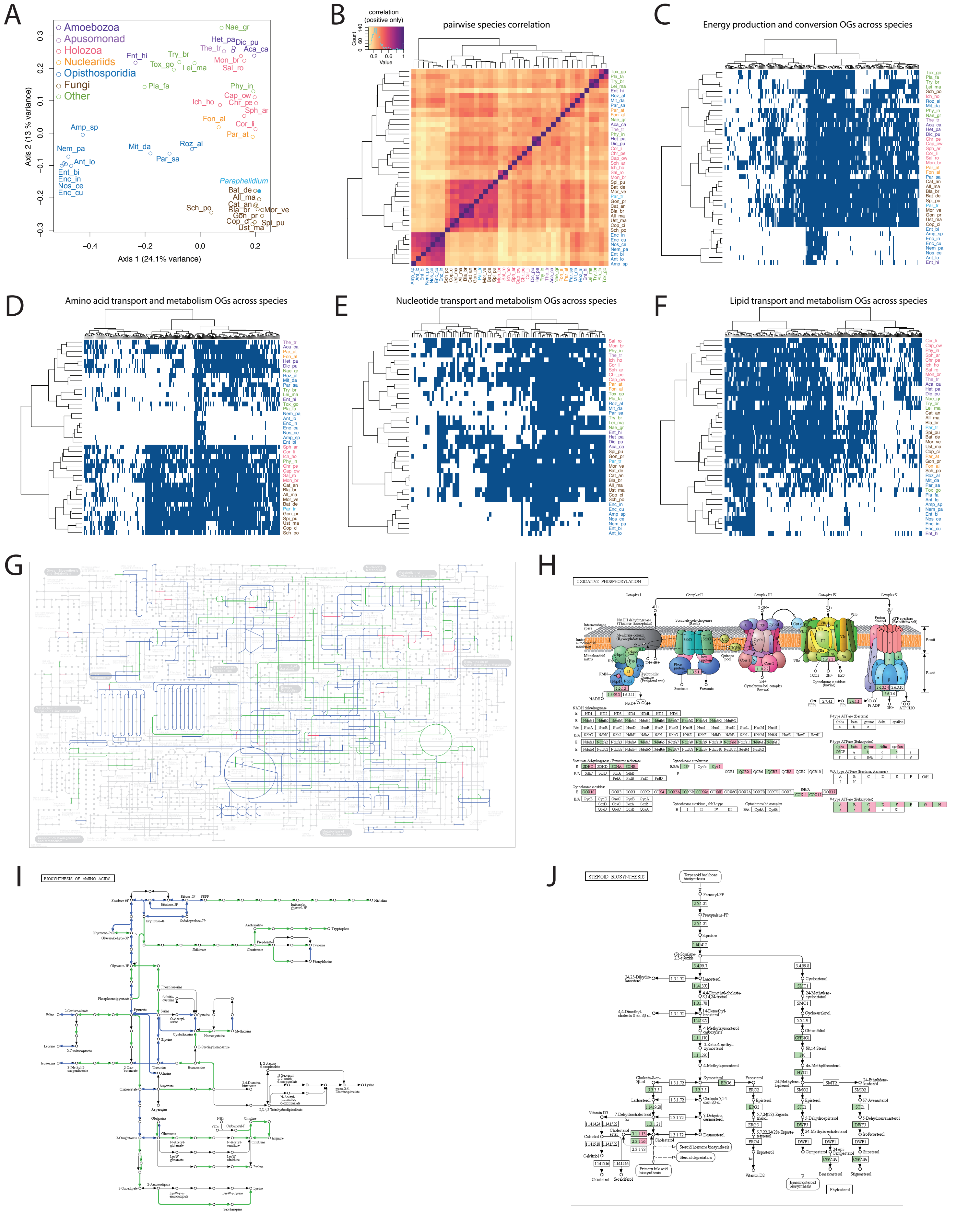

Supplementary Figure 3. Comparative analyses of metabolic gene content.

(A) Principal coordinate analysis (PCoA) of 41 eukaryotic species according to their profile of presence/absence of 1172 ortholog groups (OGs) related to 8 primary metabolic functions (COG categories C,E, F, G, H, I, P and Q); simplified version in Figure 3A. (B) Clustering of 41 eukaryotic species according to their profile of presence/absence of the same primary metabolism 1172 OGs. Color code is scaled to display positive Pearson's r (0 to 1). (C-F) Binary heat maps of orthologs (OGs) in 41 eukaryotic genomes/transcriptomes. (C) Energy production and conversion (167 OGs, category C). (D) Amino acid transport and metabolism (160 OGs, category E). (E) Nucleotide transport and metabolism (73 OGs, category F). (F) Lipid transport and metabolism (166 OGs, category I). (G) KEGG metabolic pathways map01100. (H) KEGG Oxidative phosphorylation map00190. (I) KEGG Amino acid synthesis map01230. (J) KEGG Steroid biosynthesis map00100. KEGG maps compare presence/absence of the corresponding proteins in Paraphelidium tribonemae (green) and Rozella allomycis (pink); both (blue). Full names for species acronyms are provided in supplementary text.

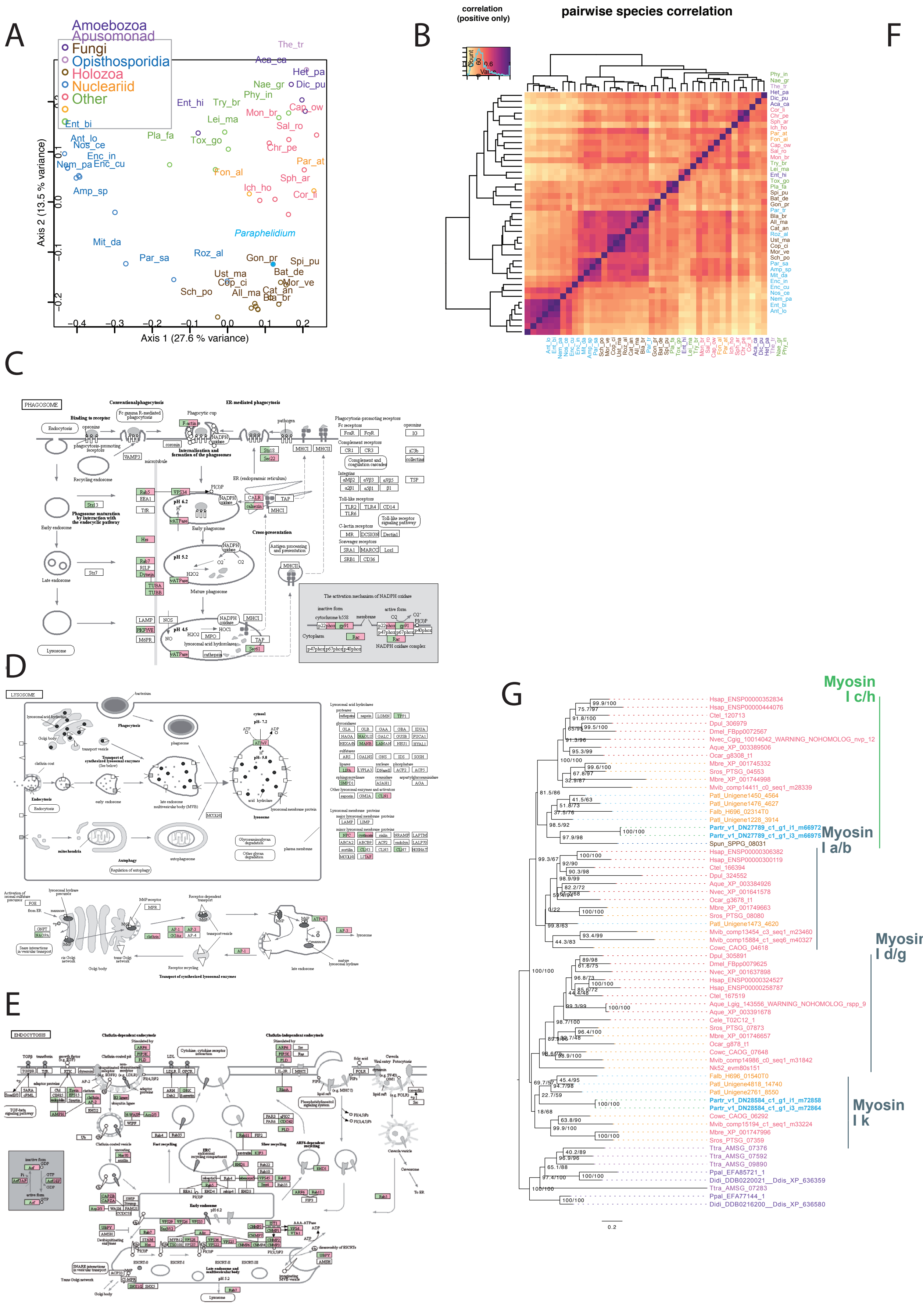

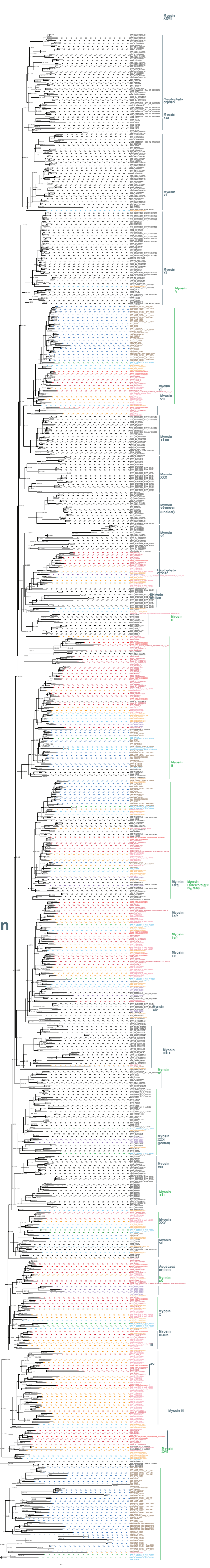

Supplementary Figure 4. Comparative analyses of proteins related to cytoskeleton, membrane trafficking and phagotrophy (A) Principal coordinate analysis (PCoA) of 41 eukaryotic species according to their profile of presence/absence of 695 KEGG groups of orthologs (KOGs) related to Cytoskeleton (KO04812), membrane trafficking (KO04131) and phagotrophy (ko04071, ko04142, ko04144, ko04145, map03050, map04130, map04141, map04146 and map04810; see Table S3); simplified version in Figure 3C. (B). Clustering of 41 eukaryotic species according to their profile of presence/absence of the same 695 OGs as in (A). Color code is scaled to display positive Pearson's r (0 to 1). (C-E) KEGG maps compare presence/absence of the corresponding proteins in Paraphelidium tribonemae (green) and Rozella allomycis (pink). (C) KEGG Phagosome map04145. (D) KEGG Lysosome map04142. (E) KEGG Endocytosis map04144. (F-G) Phylogenetic tree of myosin genes using the Myosin head domain (Pfam PF00063), using Maximum likelihood in IQ-TREE (supports are SH-like approximate likelihood ratio test / UFBS, respectively) based in the classification by Sebé-Pedrós et al. (2014). (F) Myosin class I phylogeny. (G) All eukaryotic myosins. Species are color-coded according to taxonomy.
