# Supplementary material for: Global transcriptome analysis of the aphelid Paraphelidium tribonemae supports the phagotrophic origin of fungi
Source: Commun Biol. 2018 Dec 19;1:231. doi: 10.1038/s42003-018-0235-z (PMC6299283; doi:10.1038/s42003-018-0235-z)
Supplement: Supplementary file 2 — Description of Additional Supplementary Files [file 42003_2018_235_MOESM2_ESM.docx]

**Description of Additional Supplementary Files**

**File Name**: Supplementary Data 1

**Description**: Supplementary Table 1 (excel file). eggNOG annotation of the Paraphelidium tribonemae predicted proteome.

**File Name**: Supplementary Data 2

**Description**: Supplementary Table 2 (excel file). Statistical support and alternative topology tests of phylogenomic analyses. Summary of bayesian (BI) and Maximum Likelihood (ML) phylogenomic results for each of the datasets combining three amino acid matrices (SCPD, 93 single copy protein domains (Torruella et al 2015); BMC, 53 conserved microsporidian genes (Capella-Gutiérrez et al, 2012); GBE, 259 Amphiamblysprotein markers (Mikhailov et al, 2016)) with two taxon samplings (49 species -49sp- including long-branch Microsporidia and36 species -36sp- 36sp without them). IQ-tree output alternative topology tests; and progressive exclusion of fastest evolving sites. A+F = Aphelida (Paraphelidium) + Fungi; O = Opisthosporidia; A+B = Aphelida + Blastocladiomycota; A+C = Aphelida + Chytridiomycota; A+BC = Aphelida + Blastocladiomycota and Chytridiomycota. Green-shadowed cells indicate significance. The KH, SH and AU tests return p-values; a tree is rejected if its p-value < 0.05 (marked with a - sign).

**File Name**: Supplementary Data 3

**Description**: Supplementary Table 3 (excel file). Proteins involved in primary metabolism present in various eukaryotic genomes compared in this work. Primary metabolism-related proteins are based on eggNOG classification. Full species names for acronyms are listed in Methods.

**File Name**: Supplementary Data 4

**Description**: Supplementary Table 4 (excel file). Distribution of selected proteins related to cytoskeletton, trafficking and phagotrophy in different eukaryotic genomes

**File Name**: Supplementary Data 5

**Description**: Supplementary Table 5 (excel file). Myosins detected in Paraphelidium tribonemae

**File Name**: Supplementary Data 6

**Description**: Supplementary Table 6 (excel file). SCAR/WAVE and WASP detected in Paraphelidium tribonemae and closely related species.

**File Name**: Supplementary Movie 1

**Description**: Zoospores of the aphelid Paraphelidium tribonemae in motion

**File Name**: Supplementary Software 1

**Description**: File containing all phylogenetic trees mentioned in this study in Newick format
